# CCL7 as a novel inflammatory mediator in cardiovascular disease, diabetes mellitus, and kidney disease

**DOI:** 10.1186/s12933-022-01626-1

**Published:** 2022-09-15

**Authors:** Ting-Ting Chang, Ching Chen, Jaw-Wen Chen

**Affiliations:** 1 Department and Institute of Pharmacology, School of Medicine, National Yang Ming Chiao Tung University, Taipei, Taiwan; 2 Healthcare and Services Center, Taipei Veterans General Hospital, Taipei, Taiwan; 3 Division of Cardiology, Department of Medicine, Taipei Veterans General Hospital, Taipei, Taiwan; 4 Cardiovascular Research Center, National Yang Ming Chiao Tung University, Taipei, Taiwan

**Keywords:** Cardiovascular disease, Chemokine, Chemokine CC motif ligand 7, Chronic kidney disease, Diabetes mellitus, Diabetic kidney disease

## Abstract

Chemokines are key components in the pathology of chronic diseases. Chemokine CC motif ligand 7 (CCL7) is believed to be associated with cardiovascular disease, diabetes mellitus, and kidney disease. CCL7 may play a role in inflammatory events by attracting macrophages and monocytes to further amplify inflammatory processes and contribute to disease progression. However, CCL7-specific pathological signaling pathways need to be further confirmed in these chronic diseases. Given the multiple redundancy system among chemokines and their receptors, further experimental and clinical studies are needed to clarify whether direct CCL7 inhibition mechanisms could be a promising therapeutic approach to attenuating the development of cardiovascular disease, diabetes mellitus, and kidney disease.

## Background

Chemokines are small-molecular-weight chemotactic cytokines that are secreted by several cells, such as endothelial cells, fibroblasts, neutrophils, and macrophages. They can be broadly divided into four subfamilies: CC, C, CXC, and CX3C [1, 2]. Chemokines have complex signaling pathways because they often have shared and specific chemokine receptors. In other words, some chemokines bind to multiple receptors, and receptors can share multiple chemokines from the same subfamily. Chemokines are defined as homeostatic or inflammatory depending on their characteristics. Homeostatic chemokines are constitutively secreted and are mainly involved in lymphocyte traffic, while inflammatory chemokines are related to pro-inflammatory mechanisms and induce leukocyte recruitment to augment disease [3]. Circulating chemokines may identify individuals with clinically significant cardiovascular disease [4]. Although preclinical studies have revealed the importance of several chemokines in disease, chemokine-based therapy is not yet available for clinical use in cardiovascular disease, diabetes mellitus (DM), and kidney disease [5, 6]. The potential reasons for this are poor target selection, the inappropriate timing of administration, and a deficient understanding of the complex communication system among chemokines and their receptors. If possible, the role of each chemokine and receptor should be fully and directly investigated to ensure potential clinical implementation in each individual disease.

## Chemokine CC motif ligand 7 (CCL7)

Chemokine CC motif ligand 7 (CCL7), also known as monocyte chemotactic protein (MCP)-3, is a chemotactic factor for monocytes and neutrophils. There are four kinds of MCPs (MCP-1/CCL2, MCP-2/CCL8, MCP-3/CCL7, and MCP-4/CCL13) in the CC chemokine subfamily, with 56–71% amino acid sequence homology between the four MCP chemokines [7]. The ability of bindarit to inhibit MCP production by monocytes and endothelial cells may underlie the drug’s anti-inflammatory activity in disease [8]. Though they share high similarity in the amino acid sequence, MCP chemokines have different receptors and biological activities. The human *CCL7* gene is located on chromosome 17q11.2-12 [9]. Human CCL7 proteins are synthesized as 99-amino-acid precursors. Mature protein of 76 amino acids is secreted after cleavage of the signal peptide [10]. CCL7 adopts an alphabeta fold structure [11]. CCL7 was first characterized from the human osteosarcoma supernatant [12]. Therefore, most CCL7-related studies focus on its role in tumorigenesis. Some studies indicate that CCL7 can promote tumor invasion and metastasis; however, other studies suggest that CCL7 has tumor suppressor effects [13].

CCL7 can be induced in several cell types, such as endothelial cells, vascular smooth muscle cells (VSMCs), and myelomonocytic cells, under the stimulation of phorbol 12-myristate 13-acetate [14], tumor necrosis factor (TNF)-α [15], or lipopolysaccharides [16]. In a clinical setting, higher CCL7 levels are observed in subjects with an elevated body mass index [17]. CCL7 is dominantly expressed in preadipocytes compared to adipocytes [18]. Enhanced levels of CCL7 are observed in M1 and M2a macrophages [19]. Platelet-derived growth factor-BB stimulates CCL7 expression in perivascular precursor cells and leads to increased accumulation of macrophages [20]. Moreover, CCL7 expression decreases following stimulation of interleukin (IL)-4 and IL-10 and increases following stimulation of IL-1 and TNF-α [21].

**Fig. 1:**
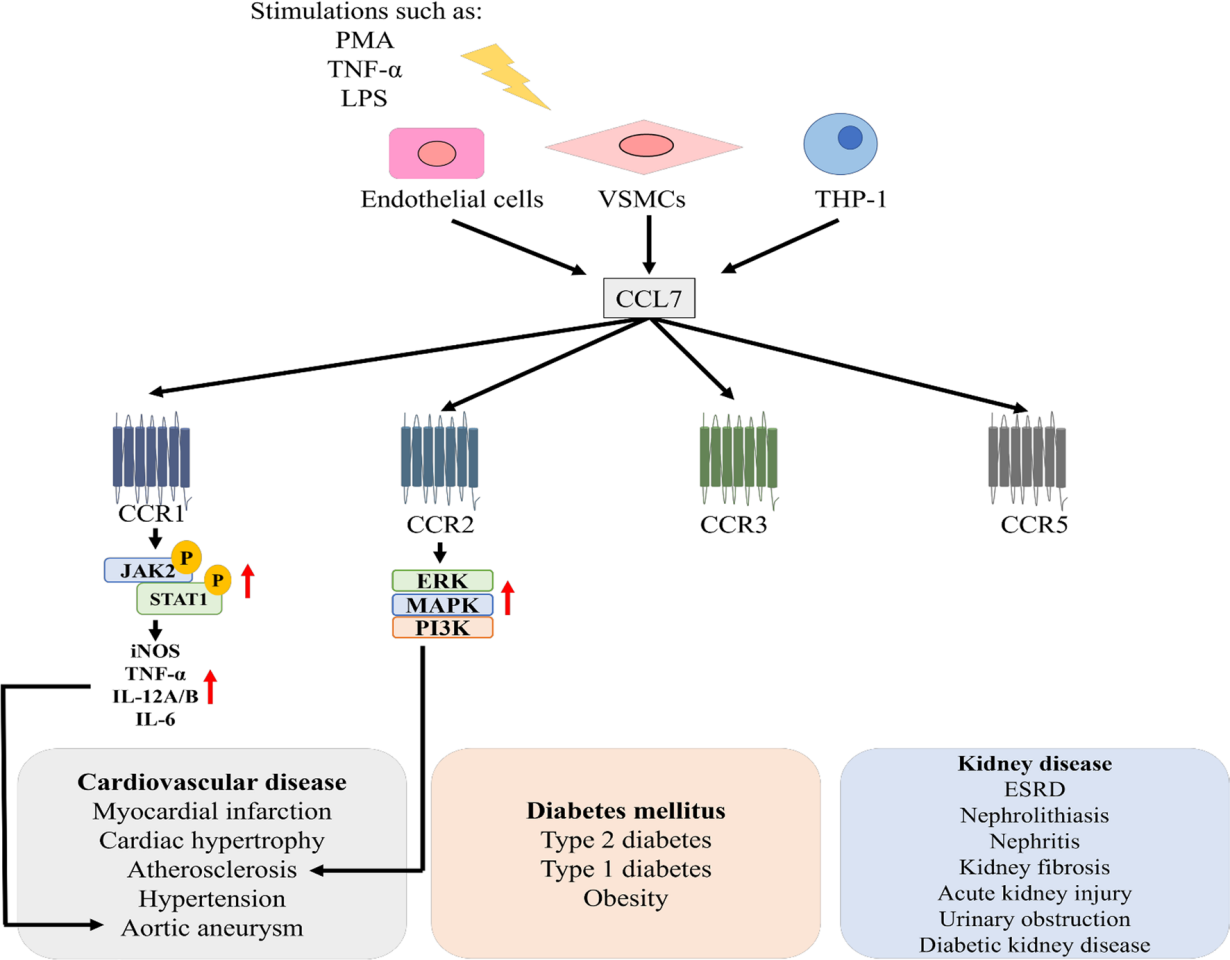
The correlation of CCL7 in cardiovascular disease, diabetes mellitus, and kidney disease in this review article. CCL7 can be induced in endothelial cells, VSMCs, and THP-1 under the stimulation of PMA, TNF-α or LPS. CCR1, CCR2, CCR3 and CCR5 are the functional receptor of CCL7. CCL7 can recruit immune cells that express associated receptors, which may result in inflammatory related disease, such as cardiovascular disease, diabetes mellitus, and kidney disease. CCR1: CC chemokine receptor 1; CCR2: CC chemokine receptor 2; CCR3: CC chemokine receptor 3; CCR5: CC chemokine receptor 5; ERK: extracellular signal-regulated kinases; ESRD: *end-stage renal disease;* iNOS: inducible nitric oxide synthase; IL-6: interleukin-6; IL-12A/B: interleukin-12A/B; JAK2: Janus kinase 2; LPS: lipopolysaccharides; *MAPK: mitogen-activated protein kinases;* PI3K: phosphoinositide 3-kinases; PMA: phorbol 12-myristate 13-acetate; *STAT1: signal transducer and activator of transcription 1;* THP-1: myelomonocytic cells; *TNF-α: tumor necrosis factor-alpha;* VSMCs: vascular smooth muscle cells

As mentioned previously, chemokines and their receptors often have complex networks, that is, they bind to multiple receptors. CC chemokine receptor (CCR)1, CCR2, CCR3, and CCR5 are known as the functional receptors of CCL7. CCR1 and CCR3 may cause directional migration of circulating angiogenic cells; CCR2 and CCR5 are most critical to the monocyte mobilization [22, 23]. Among them, CCR2 is the most well-known receptor of MCPs. MCP chemokines can stimulate CCR2, which is located on monocytes and macrophages and is associated with the pathogenesis of atherosclerosis and type 2 DM [24, 25]. Both exogenous and endogenous CCL7 can recruit leukocytes that express associated receptors to migrate along the concentration gradient to the sites of inflammation. In monocyte mobilization from bone marrow to blood circulation, CCR2 and CCL7 are critical for the recruitment of monocytes to sites of inflammation [26]. It is indicated that CCL7 may be also involved in the development of aortic aneurysm via the CCR1-related mechanisms [27]. However, the individual role of CCR3 and CCR5 in the in vivo effects of CCL7 still await further clarified (Fig. 1).

Decreased CCL7 levels may result in a loss of their chemotactic effects on leukocytes and a subsequent reduction in inflammatory cell recruitment, while elevated CCL7 levels may contribute to increased inflammation. Though both preclinical studies and clinical trials show the beneficial effects of CCR2 inhibition, especially in atherosclerosis, no drugs have been approved as yet [28]. One major possibility is that our understanding of the multiple redundancy system among chemokines and their receptors is still deficient, which may hinder potential translation to clinical applications. Accordingly, the role of CCL7 itself should be further confirmed, especially in inflammatory-related diseases. This review focuses on the emerging evidence on the roles of CCL7-related mechanisms in both experimental and clinical cardiovascular disease, DM, and kidney disease. It is aimed to provide a rationale for the potential role of CCL7-related mechanisms as therapeutic targets in these diseases.

## The potential role of CCL7 in cardiovascular disease

### Myocardial infarction and cardiac hypertrophy

Most CCL7-related studies on cardiovascular disease have been performed in myocardial infarction. Elevated circulating CCL7 levels are observed in patients with acute myocardial infarction and can be used as a predictor of the risk of death or recurrent myocardial infarction [29]. Increased CCL7 levels are detected in local extracellular vesicle generation in the infarcted heart coordinates of cardiac inflammation after myocardial infarction [30]. Upregulated CCL7 expressions are observed in rat hearts after ischemia and participate in the recruitment of CD34^+^ bone marrow progenitor cells to the ischemic myocardium [31]. In addition, CCL7 may be a myocardial mesenchymal stem cell (MSC) homing factor. CCL7 upregulation can help recruit MSCs to the injured areas and improve cardiac remodeling [32]. Overexpression of LIM-homeobox transcription factor islet-1 in MSCs promotes angiogenesis by increasing CCL7 secretion and enhancing the survival of human umbilical vein endothelial cells [33]. On the contrary, MSC application in Coxsackievirus B3-induced myocarditis reduces myocardial inflammation with decreased left ventricular CCL7 mRNA expression [34]. In rodent models of cardiac hypertrophy, left ventricular CCL7 expression is enhanced in the early inflammatory phase [35, 36]. This discussion indicates that the role of CCL7 is still undefined in cardiovascular disease.

### Angiogenesis

Importantly, low CCL7 levels are observed in culture supernatants of circulating angiogenic cells but relatively high levels are observed in culture supernatants of macrophages, suggesting that the CCL7-induced migration of circulating angiogenic cells is majorly via the paracrine mechanism. In vivo, CCL7 can induce blood vessel formation in Matrigel plugs in normal 8- to 11-week-old C57BL6 mice under physiological conditions [37]. Notably, aging could upregulate aortic CCL7 expression in wild-type mice [38]. Collectively, these data imply that CCL7 might promote angiogenesis at low concentrations (physiological or young conditions) and attenuate angiogenesis at relatively high concentrations (pathological or elderly conditions). Although the pathological role of CCL7 is unknown, more direct and solid evidence is needed.

### Atherosclerosis

Cytokine-induced CCL7 expression is enhanced in smooth muscle cells (SMCs) and in the carotid artery after balloon angioplasty, indicating the potential role of CCL7 in the pathogenesis of restenosis and atherosclerosis [39]. In a mouse model of atherosclerosis, low-density-lipoprotein (LDL)-receptor-deficient mice fed a high-fat diet showed enhanced arterial thrombosis with increased plasma CCL7 expression and altered gut microbial diversity [40]. In addition, CCL7 can promote human coronary artery SMC and VSMC proliferation in vitro [41, 42]. In CCL7-overexpressing transgenic mice fed a high-fat-and-high-cholesterol diet, higher plasma total cholesterol levels and higher lipid accumulation in the aorta were observed compared to wild-type mice [43]. Further, CCL7 expression can be induced by oxidized LDL in human monocytic THP-1 cells in vitro [44]. Although the absence of proprotein convertase subtilisin/kexin type 9 in atherosclerosis-prone Ldlr^−/−^Apobec1^−/−^ mice esulted in decreased lipid and apoB levels, fewer atherogenic LDLs, and reduced atherosclerosis, the LDLs from these mice could induce lower endothelial expression of intercellular adhesion molecule-1, CCL2, CCL7, IL-6, and IL-1β [45]. Altogether, these data seem to indicate that CCL7 is not only a biomarker but also one of the potential contributors to atherosclerosis, especially in the early stages. A CCL7-deficient animal model may be required to confirm the direct role of CCL7 in the development as well as progression of atherosclerosis.

### Hypertension and aortic aneurysm

CCL7 inhibition with antibodies can attenuate angiotensin-II-induced hypertension and vascular remodeling, accompanied by decreased macrophage infiltration [46]. In a deoxycorticosterone acetate/salt-induced hypertension mouse model, mRNA expression of CCR2 and its ligands, such as CCL2, CCL7, CCL8, and CCL12, in the aorta was upregulated [47]. In patients with pulmonary arterial hypertension, upregulated CCL7 is associated with unfavorable 5-year transplant-free survival rates [48]. In a mouse model of renovascular hypertension, Ccl2-deficient mice show reduced mononuclear cell infiltration and decreased *Ccl7* gene expression [49]. Furthermore, aging can lead to increased renal and aortic CCL7 expression in wild-type mice [38]. CCL7 leads to angiotensin-II-induced abdominal aortic aneurysm by promoting the M1 phenotype of macrophages through the CCR1/JAK2/STAT1 signaling pathway [27]. Protein-kinase-C-δ-knockout mice show attenuated inflammation in models of abdominal aortic aneurysm with reduced CCL7 expression in the abdominal aortic artery [50]. Taken together, it seems that CCL7 is inducible and may be involved in the pathological process of hypertension and vascular remodeling. Importantly, it may play an amplifying role in the inflammation process by attracting macrophages and monocytes. Nevertheless, the specific signaling pathways related to CCL7 regulation need further investigation.

## The potential role of CCL7 in type 1 and type 2 diabetes mellitus (DM)

### Type 1 DM

Acute hyperglycemia can result in upregulated urinary expression of CCL7 in patients with type 1 DM [51]. Cardiac diastolic abnormality and CCL7 were also independently associated in a subset of young type 1 diabetic patients during acute diabetic ketoacidosis, suggesting the potential link of CCL7-related systemic inflammation with the presence of cardiac diastolic dysfunction [52]. Furthermore, in a mouse model of type 1 DM, T lymphocyte exosomes may induce CCL7 in β cells to promote the recruitment of immune cells and exacerbate β cell death [53]. Chemokine signaling pathways related to CCL2, CCL7, and CXCL10 could be involved during β cell apoptosis in type 1 DM animal models with non-obese diabetic mice [54]. In a cyclophosphamide-induced non-obese diabetic mouse model, an increase in the transcripts of chemokine genes, such as *CXCL1*, *CXCL5*, and *CCL7*, was observed in purified islets [55]. CCL7 may be one of the contributors to islet damage in the development of type 1 DM.

### Type 2 DM

CCL7 levels could be upregulated in patients with type 2 DM [56]. In type 2 diabetic obese patients, adipose interferon regulatory factor 5 transcripts were positively associated with CCL7 [57]. Patients with type 2 DM and ischemic stroke had higher serum CCL7 levels than patients with ischemic stroke alone [58]. Although the recruitment of immune cells to adipose tissue is altered in obesity, which causes insulin resistance in type 2 DM, the dominant factor for recruiting macrophages in adipose tissue during obesity should be further defined [59]. Interestingly, in mice with severe combined immunodeficiency on a normal chow diet, insulin resistance was associated with increased CCL7 levels [60]. Moreover, a high-fat diet increases neutrophil infiltration into adipose tissue with upregulated CCL7 levels [61], suggesting its potential harmful role in metabolic syndrome. Altogether, CCL7 may be involved in the development of adipocyte inflammation and insulin resistance in type 2 DM. However, the underlying mechanism is unknown, and further experiments are required to confirm this hypothesis.

## The potential role of CCL7 in kidney disease

### End-stage renal disease, nephritis, nephrolithiasis, and acute kidney injury

Previous studies have shown the relationships between CCL7 and kidney damage in different models of kidney diseases, such as kidney injury, glomerulonephritis, kidney stone, end-stage renal disease and so on. End-stage renal disease patients exhibit Mycobacterium-tuberculosis-specific CCL7 expression in the absence of interferon-γ [62]. CCL7 expression is markedly enhanced in the papillary tips, kidney urine, and bladder urine of patients with nephrolithiasis [63]. Transplantation of the human-*OXR1*-gene-integrated MSCs significantly reduced macrophage and T lymphocyte infiltration by decreasing CCL7, IL-1β, IL-6, and NF-κB expression in the injured kidneys of a nephritis mouse model [64]. CCL7 can be produced from activated primary-cultured mesangial cells from lupus nephritis mice [65]. In a rat model of puromycin-aminonucleoside-induced nephrosis, CCL7 mRNA levels increased on day 5 and returned to normal by day 7 in the renal cortex [66].

CCL7 could be upregulated by transforming growth factor-β1, which plays a premier role in kidney fibrosis in NRK-49F normal rat kidney fibroblasts [67]. The blockade of the proinflammatory kinin B1 receptor has antifibrotic effects by inhibiting CCL7 expression [68]. In response to acute kidney injury, B cells produce CCL7 to facilitate neutrophil and monocyte recruitment to the injured sites [69]. Treatment with oxalate upregulates CCL7 expression in human renal proximal tubular epithelial cells [70]. Oncostatin M is upregulated in the early phases of urinary obstruction. Oncostatin M overexpression in tubular epithelial cells leads to epithelial-myofibroblast trans-differentiation, and oncostatin M treatment upregulates CCL7 mRNA in kidney fibroblasts [71]. A previous study has suggested the dual role of CCL7 in the development of kidney tubular interstitial fibrosis, deleterious in early stages but beneficial in later stages in a model of unilateral ureteral obstruction [72]. The administration of the p38 inhibitor SB203580 blocked CCL7 induction in the cuffed kidneys in a model of renal artery stenosis [73]. Therefore, CCL7 may contribute to the development of kidney fibrosis in different types of kidney diseases.

### Diabetic kidney disease (DKD)

Kidney failure is one of the major complications from DM. In the unique pathology of DKD, changes in the glomerular structure, including mesangial expansion, reduction in the capillary surface, and podocyte loss, are major features [74]. Inflammation is considered a novel mechanism linked to DKD progression. Macrophage accumulation and infiltration play a key role in DKD development through the production of reactive oxygen species, cytokines, and proteases. Moreover, kidney fibrosis results from extracellular matrix deposition, which is caused by the infiltration of immune cells, inflammatory cells, and altered chemokines and cytokines in the kidney [75]. However, the mechanistic role of chemokines including CCL7 in clinical DKD is not well clarified [6]. While CCL7 may be known as a proinflammatory chemokine related to kidney fibrosis progression in other chronic kidney diseases, its direct mechanistic role remains unclear in DKD. Limited clinical data suggest that acute hyperglycemia may cause upregulated urinary CCL7 expression in patients with type 1 DM [51]. Future studies may be required to explore the potential impacts of chemokines such as CCL7 on DKD in this regard.

## Conclusion

Elevated CCL7 expression is observed in cardiovascular disease (Table 1), DM (Table 2), and kidney disease (Table 3). However, the current CCL7-related studies were conducted by different models in different species. Given the limited information, it may be difficult to define the baseline expression of mRNA and protein in these diseases. Future research is required to clarify these issues. While the detailed pathological role of CCL7 and related signaling pathways in these diseases need further confirmation, it has been suggested that CCL7 may promote the progression of atherosclerosis and aortic aneurysm and play a significant role in the inflammatory events underlying most vascular diseases, DM, and kidney disease by attracting macrophages and monocytes to amplify inflammatory processes and contribute to the disease progression (Fig. 1). Given the specific pathological background in each individual disease model, the mechanics of CCL7 should be fully investigated to ensure translation to clinical trials. Some previous studies suggested the potential beneficial effects of CCL7 inhibition by neutralizing antibody or genetic knockout in myocardial infarction, atherosclerosis, aortic aneurysm, acute kidney injury, and in later stages of unilateral ureteral obstruction [27, 29, 42, 69, 72]. However, there are currently no target drugs or small molecule drugs against CCL7. Due to the multiple redundancy system among chemokines and their receptors, further experimental and clinical studies should be interesting to focus on direct anti-CCL7 mechanisms as a promising therapeutic approach to attenuating the development of cardiovascular disease DM, and kidney disease.

**Table 1:** Summary of the CCL7 in cardiovascular disease in this review article

Cardiovascular disease			References
Myocardial infarction	CCL7↑	Risk of death↑/Recurrent myocardial infarction↑/Cardiac inflammation↑	[29–31]
	CCL7 (MSCs) ↑	Cardiac remodeling↑ /Survival of HUVECs ↑/Myocardial inflammation↑	[32–34]
Cardiac hypertrophy	CCL7↑	Inflammation (Early phase of cardiac hypertrophy) ↑	[35, 36]
Angiogenesis	CCL7↑	Formation of blood vessels (Physiological condition) ↑	[37, 38]
Atherosclerosis	In VSMCs and in carotid artery after balloon angioplasty CCL7↑ CCL7 (monocyte) ↑	Arterial thrombosis↑/-altered gut microbial diversity/VSMCs proliferation↑/Lipid accumulation in the aortas↑/ Endothelial expression of ICAM-1, CCL2, CCL7, IL-6, and IL-1β↑	[39–45]
Hypertension	CCL7↑	5-year transplant-free survival rates↓/Mononuclear cell and macrophage infiltration↑	[46–49]
Aortic aneurysm	CCL7↑	M1 phenotype of macrophage (CCR1/JAK2/STAT1) ↑	[27, 50]

*HUVECs* human umbilical vein endothelial cells, *ICAM-1* intercellular adhesion molecule-1, *IL-1β: interleukin-1β*, IL-6: *interleukin-6*, *MSCs* mesenchymal stem cells, *VSMCs* vascular smooth muscle cells

**Table 2:** Summary of the CCL7 in diabetes mellitus in this review article

Diabetes mellitus			References
Type 1 diabetes	CCL7 (T lymphocyte exosomes) ↑	Recruitment of immune cells↑/β-cell apoptosis ↑/Diastolic abnormality	[51–54]
	CCL7 (Islets cells) ↑	CXCL1, CXCL5, and CCL7 ↑	[55]
Type 2 diabetes	CCL7 (Adipose tissue) ↑	Interferon regulatory factor 5 transcripts were positively associated with CCL7	[56, 57]
		Neutrophil infiltration↑/Insulin resistance↑	[59–61]
Diabetes and ischemic stroke	Serum CCL7 ↑		[58]

**Table 3:** Summary of the CCL7 in kidney disease in this review article

Kidney disease			References
ESRD	Exhibit mycobacterium tuberculosis-specific CCL7 expression		[62]
Nephrolithiasis	CCL7 in papillary tips, kidney urine, and bladder urine ↑		[63]
Nephritis	CCL7 ↑	Macrophage and T lymphocyte infiltration ↑	[64–66]
Kidney fibrosis	CCL7 ↑	CCL7 could be up-regulated by TGF-β1	[67, 68]
Acute kidney injury	CCL7 (B cell) ↑	Facilitate neutrophil and monocyte recruitment ↑	[69]
Kidney stone	Oxalate upregulates CCL7 expressions in the human renal proximal tubular epithelial cells		[70]
Urinary obstruction	Oncostatin M treatment upregulates CCL7 mRNA in kidney fibroblasts/ kidney tubular interstitial fibrosis ↑		[71–73]
Diabetic kidney disease	Urinary expressions CCL7 in type 1 diabetes ↑		[51]

*ESRD end-stage renal disease*, *TGF-β* transforming growth factor-β

## Data Availability

Not applicable.

## Abbreviations

CCL7: Chemokine CC motif ligand 7
CCR: Chemokine receptor
DKD: Diabetic kidney disease
DM: Diabetes mellitus
IL: Interleukin
LDL: Low-density-lipoprotein
MCP: Monocyte chemotactic protein
MSC: Mesenchymal stem cell
SMC: Smooth muscle cell
TNF: Tumor necrosis factor
VSMC: Vascular smooth muscle cell
